# Neoadjuvant chemoradiotherapy in combination with deep regional hyperthermia followed by surgery for rectal cancer: a systematic review and meta-analysis

**DOI:** 10.1007/s00066-024-02312-9

**Published:** 2024-10-17

**Authors:** Adela Ademaj, Sonja Stieb, Cihan Gani, Oliver J. Ott, Dietmar Marder, Roger A. Hälg, Susanne Rogers, Pirus Ghadjar, Rainer Fietkau, Hans Crezee, Oliver Riesterer

**Affiliations:** 1 Centre for Radiation Oncology, Cantonal Hospital Aarau, 5001 Aarau, Switzerland; 2 Doctoral Clinical Science Program, Medical Faculty, University of Zürich, 8032 Zürich, Switzerland; 3 Department of Radiation Oncology, Universitätsklinikum Tübingen, 72076 Tübingen, Germany; 4 Department of Radiation Oncology, Universitätsklinikum Erlangen, 91054 Erlangen, Germany; 5 Comprehensive Cancer Center Erlangen-EMN, 91054 Erlangen, Germany; 6 Institute of Physics, Science Faculty, University of Zürich, 8057 Zürich, Switzerland; 7 Department of Radiation Oncology, Charité—Universitätsmedizin Berlin, Freie Universität Berlin, Berlin Institute of Health, Humboldt-Universität zu Berlin, Berlin, Germany; 8 Department of Radiation Oncology, Amsterdam UMC, Cancer Center Amsterdam, University of Amsterdam, 1105 AZ Amsterdam, The Netherlands

**Keywords:** Chemoradiotherapy, Deep regional hyperthermia, Locally advanced rectal cancer, Locally recurrent rectal cancer

## Abstract

**Background and purpose:**

Combining chemoradiotherapy (CRT) with deep regional hyperthermia (HT) shows promise for enhancing clinical outcomes in selected rectal cancer patients. This study aimed to integrate the evidence and evaluate the efficacy of this combined treatment approach.

**Materials and methods:**

A systematic search of the PubMed, Scopus, and Mendeley databases was performed. This review was conducted according to the PRISMA guidelines. The quality of studies was evaluated using the Newcastle–Ottawa scale (NOS). Random-effects meta-analyses (DerSimonian and Laird) were performed. The primary outcome was pathological complete response (pCR), and secondary endpoints were overall survival (OS), disease-free survival (DFS), local recurrence-free survival (LRFS), and toxicity.

**Results:**

In total, 12 studies were included, mostly of moderate quality. Patients with locally advanced rectal cancer (LARC; *n* = 760) and locally recurrent rectal cancer (LRRC; *n* = 22) were eligible. The pooled pCR rate was 19% (95% confidence interval [CI]: 16–22%) among all 782 patients and 19% (95%CI:16–23%) among 760 LARC patients. Due to significant study heterogeneity, survival outcomes were pooled by excluding LRRC patients. The pooled 5‑year OS rate among 433 LARC patients was 87% (95%CI: 83–90%). The pooled 5‑year DFS and LRFS in LARC patients were 75% (95%CI: 70–80%) and 95% (95%CI: 92–97%), respectively. There was a lack of consistent reporting of HT treatment parameters and toxicity symptoms among the studies.

**Conclusion:**

The collective clinical evidence showed that neoadjuvant CRT combined with HT in rectal cancer patients is feasible, with a 19% pCR rate and excellent survival outcomes in long term follow-up.

**Supplementary Information:**

The online version of this article (10.1007/s00066-024-02312-9) contains supplementary material, which is available to authorized users.

## Introduction

The standard preoperative radiotherapy schedule for patients with locally advanced rectal cancer (LARC) is either chemoradiotherapy (CRT) with 50–56 Gy delivered over 4–5 weeks or short-course radiotherapy with 5 × 5 Gy given in one week [1–3]. With the aim of achieving organ preservation and improving quality of life, a watch-and-wait strategy has recently been broadly introduced to selected patients for whom a clinical complete response has been achieved after either CRT [4] or total neoadjuvant treatment (TNT) consisting of radiotherapy and either induction or consolidation chemotherapy [5] or escalation of the radiotherapy dose [6]. To further improve the rates of local control and of clinical or pathological complete response (pCR), novel treatment strategies are currently being explored.

The potential of deep regional hyperthermia (HT) to improve the local effect of CRT in patients with rectal cancer has recently been shown in two German phase II studies [7, 8]. Notably, these studies were designed in the pre-TNT era. In the German HyRec phase II trial by Ott et al. [7], a preoperative radiation regimen of 45–50.4 Gy in combination with HT, 5‑fluorouracil (5-FU)/capecitabine, and oxaliplatin was shown to be a feasible treatment for LARC and locally recurrent rectal cancer (LRRC) patients. The HyRec treatment regimen resulted in a comparatively high pCR rate of 19% (20/105) for LARC and LRRC patients if compared to 8% pCR in the landmark CAO/ARO/AIO-94 trial that established preoperative CRT as a standard treatment [9], including the standard arms of recent phase III randomized TNT clinical trials such as RAPIDO (12% pCR) [2] or PRODIGE-23 (14% pCR) [10]. The second German phase II study performed by the Tübingen research group investigated 5‑FU-based preoperative radiochemotherapy with 50.4 Gy in 28 fractions combined with HT and reported 3‑year overall survival (OS), disease-free survival (DFS), and local and distant control rates of 94%, 81%, 96%, and 87%, respectively [8]. Additionally, a previous retrospective analysis from the same research group showed that the rate of pCR was significantly higher (16.4%) for patients treated with CRT in combination with at least four HT sessions in comparison to patients treated with CRT only (6.7%) [11]. Recently, the long-term clinical outcomes of a phase II study conducted in Norway on LARC and LRRC patients treated with HT and CRT were published [12]. Schem et al. reported improved 5‑ and 10-year cancer-specific survival outcomes of 73.5% and 62.5% and a high pCR rate of 30% (14/47) [12]. These results confirmed the outcomes reported in an Italian phase II study which reported a high pCR rate of 24% (18/76) after neoadjuvant CRT combined with HT [13]. However, further clinical evidence is required to assess the efficacy of HT in combination with CRT to increase the rate of pCR or even the organ-preservation rate for LARC or LRRC patients in randomized clinical trials. In trials exploring TNT and watch-and-wait strategies in LARC and early rectal cancer patients treated with a brachytherapy boost, the preservation rates at 3 years were high, at up to 53% and 81%, respectively [5, 6]. HT has not been integrated into the TNT treatment strategy so far.

As several reports of HT in combination with preoperative CRT for rectal cancer patients have now been published and because the emerging concept of watch and wait is gaining ground, it has become necessary to systematically analyze the results of these studies. Therefore, we performed a systematic review and meta-analysis of published clinical studies to evaluate the available evidence, particularly the clinical feasibility and efficacy of preoperative CRT combined with HT in rectal cancer patients.

## Materials and methods

### Literature search strategy and selection criteria

This prospective systematic review and meta-analysis was carried out according to a prespecified protocol registered in the PROSPERO database (ID: CRD42022365439). The systematic review of clinical scientific reports was performed according to the Preferred Reporting Items for Systematic Reviews and Meta-Analyses (PRISMA) recommendations (see supplementary file: Table S1) to assess the eligible studies for the planned meta-analysis [14]. Furthermore, the guidelines for reporting systematic reviews and meta-analyses of observational studies were followed [15, 16].

Two investigators (AA and SS) performed a comprehensive literature search of the PubMed, Scopus, and Mendeley databases from inception to April 21, 2023. To find the studies using CRT in combination with HT to treat rectal cancer, databases were searched electronically using the following Medical Subject Headings (MeSH): “radiochemotherapy” OR “chemoradiotherapy” AND “hyperthermia” AND “rectum cancer” OR “rectal cancer”. The full search strategy is reported in the supplementary file (Table S2).

Based on our defined inclusion criteria, clinical studies were included if they reported pCR as a clinical outcome for rectal cancer patients who were treated with neoadjuvant CRT in combination with HT. Studies that included patients treated with thermal ablation techniques; interstitial, high-intensity focused ultrasound, or whole-body HT; or patients treated with definitive CRT for rectal cancer only were excluded. In addition, we excluded studies that included pediatric rectal cancer patients, review articles, conference proceedings, correspondence, case reports or series, pilot studies, publications in abstract form only, and editorials. Only scientific publications in English were included. In the event that clinical studies included overlapping patients from the same research group, only the largest study was included.

### Data extraction and quality analysis

The abstracts of the identified studies were screened independently by the investigators (AA and SS). Those abstracts that met the inclusion criteria (neoadjuvant treatment, patients older than 18 years, radiofrequency HT technique, advanced or recurrent rectal cancer patients) underwent full-text review. When disagreements occurred between investigators, they were resolved by consensus.

The quality of the included studies was assessed by using the Newcastle–Ottawa scale (NOS) for observational studies [17, 18]. The scale evaluates three domains of bias: selection of participants, comparability, and measure of outcomes. Studies with NOS scores of 7–9 were considered to be of high quality, and NOS scores of 4–6 were considered medium quality [17, 18]. In addition, the Grading of Recommendations, Assessment, Development, and Evaluations (GRADE) system, a transparent framework for developing and presenting summaries of evidence in a systematic approach, was used to assess the quality of the pCR rate outcome across the included studies [19].

### Outcomes

The primary outcome was pCR rate. Secondary outcomes included OS, local recurrence-free survival (LRFS), DFS, and toxicity of grades 3–4.

### Statistical analysis

Descriptive statistics were reported as the median and range for continuous variables and as percentages for categorical variables. The between-study heterogeneity was taken into account using the inverse variance-weighted random-effects model (DerSimonian and Laird) [20]. The primary and secondary outcomes were presented as proportions (as event rates), with the corresponding prediction intervals. The pCR rate, as a primary outcome, was evaluated based on intention-to-treat analysis. The heterogeneity among studies was assessed using the Cochrane Q statistic and I squared (*I*^*2*^) statistics. Heterogeneity was considered to be statistically significant if the *p*-value of the Cochrane Q test was less than 0.05 and the *I*^*2*^ statistic value was higher than 50% [21]. We subsequently performed sensitivity analyses to determine the influence of individual studies on the overall effect, including a leave-one-out analysis, which iteratively removed one study at a time, generating Baujat plots, and influence diagnostics [22]. Publication bias was assessed by constructing a contour-enhanced funnel plot and by using Egger’s regression test [23]. The statistical analyses were performed in R studio (v. 4.0.5; R studio, Inc. Boston) using the “meta” and “dmetar” packages.

## Results

### Study selection and characteristics

**Fig. 1:**
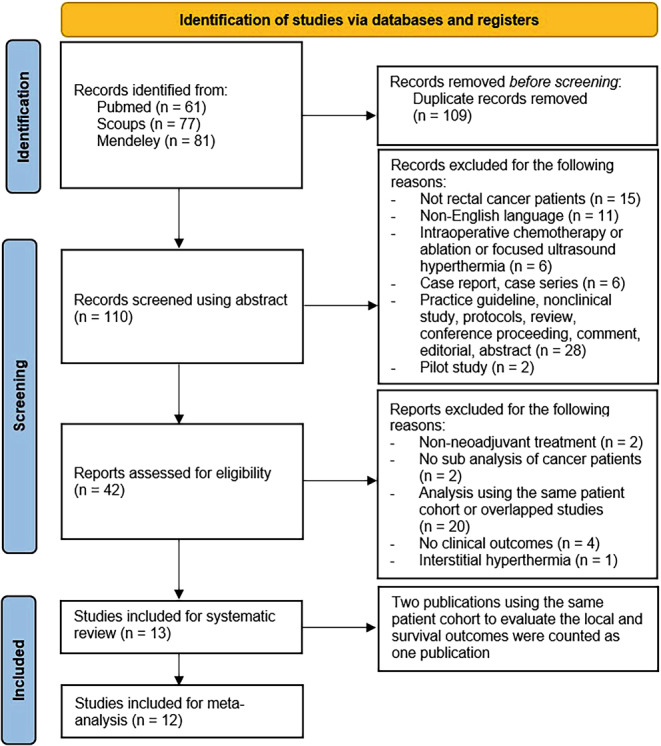
Flowchart of study selection. The number of patients (*n*) in the right-sided boxes do not add up to the number on the left because the abstracts or publications were excluded due to several reasons, as shown in right-sided boxes

Our initial search of the three databases identified 219 scientific reports and 110 abstracts were screened (Fig. 1). Forty-two studies were evaluated using both abstracts and full texts to determine whether they met the inclusion criteria. Finally, 13 studies fulfilled our inclusion criteria and were included in the systematic review. The process of study identification according to the PRISMA guidelines is summarized in Fig. 1.

Seven of the 13 included studies (54%) were nonrandomized prospective studies. The retrospective analysis performed by the Tübingen group published the survival data and the pathological complete response rates separately [11, 24]; thus, both publications counted as one study for the meta-analysis. In the study published by Kang et al. [25], patients were treated to a total radiation dose of either 39.6 Gy (group A) or 45 Gy (group B). In this analysis, the data for groups A and B were extracted and analyzed separately. General information regarding the 12 included studies is summarized in Table 1; [7, 8, 12, 13, 24–31].

**Table 1:** General information of the included studies

Author, year	Country	Study type	Comparative arm	*n*	Male/female	Median age (range)	T stage	N stage
Wang [26]	China	R	Yes	50	40/10	60.6 ± 11.6^a^	T2: 1; T3: 35; T4: 14	N0: 13; N+: 37
Schem [12]	Norway	P	No	49	32/17	59 (21–75.6)	T3: 19; T4: 24	N0: 3; N1: 17; N2: 21; Nx: 2
Lee [27]	S. Korea	P	No	60	45/15	59 (33–83)	T3: 46; T4: 14	N1: 28; N2: 32
Ott [7]	Germany	P	No	105	82/23	58 (23–85)	T2: 7; T3: 70; T4: 27	N0: 17; N1: 48; N2: 39
Gani [8]	Germany	P	No	78	46/32	63 (53–71)	T2: 5; T3: 67; T4: 6	N0: 18; N1–2: 60
Shoji [28]	Japan	R	No	81	61/20	62 (33–89)	T2: 20; T3: 45; T4: 16	N0: 40; N1: 38; N2: 2; N+: 1
Gani [24]	Germany	R	Yes	60	34/26	64.3 (38–89)	T3: 55; T4: 5	N0: 11; N+: 49
Kato [29]	Japan	R	No	48	37/11	61 (33–75)	T2: 7; T3: 37; T4: 4	n.r.
Barsukov [30]	Russia	P	No	64	40/24	57 (24–79)	T4: 64	n.r
Kang [25]	Korea	R	Yes	98	82/16	60 (18–83)	T3: 94; T4: 4	N0: 62; N+: 36
Maluta [13]	Italy	P	No	76	50/26	60 (38–82)	T3: 68; T4: 8	N0: 35; N+: 41
Rau [31]	Germany	P	No	37	31/6	59.1 (31–74)	T3: 23; T4: 14	n.r.

*R* retrospective study; *P* prospective study; *n.r.* not reported

^a^Mean value

The radiation dose and fractionation schemes, including the type of chemotherapeutic drug(s) administered and HT sessions delivered, could be extracted from all of the studies. The concurrent chemotherapy regimens differed between studies (Table 2). Furthermore, the total radiation dose ranged between 39.6 and 50.4 Gy in 22–28 fractions. Conventionally fractionated radiotherapy was delivered, except for in one study which used a hypofractionated scheme (40 Gy/10 fractions; Table 2). The total number of sessions and the HT devices were reported in all studies, but only a few studies reported the treatment sequencing, estimated mean or median values of the thermal dose expressed as cumulative equivalent minutes at 43 °C (CEM43), and temperature metrics. The treatment parameters reported in each study are summarized in Table 2. In a few studies, data regarding the surgical interventions were also provided (supplementary Table S3).

**Table 2:** Clinical information from the included studies

First author	Total RT dose (Gy)/no. of fractions	Chemotherapy drug and concentration (mg/m^2^ per day)	HT technique	Median HT session (range)	CEM43 (min)	Temperature metric (°C)	Median FU in months (range)	pCR	OS	DFS	LRFS
Wang et al. [26]	45–50/25	1650 capecitabine	Capacitive	7 (2–10)	1.10^b^ (0.34–2.34)	n.r.	49 (4–87)	22%	5‑year: 92.7%	5‑year: 61.4%	5‑year: 96.8%
Schem et al. [12]	54–56/27–28	825–1650 capecitabine + 55–100 each week oxaliplatin	Radiative	5 (1–6)	n.r.	T_avg_^b^ = 39.9 (95%CI 38.2–41.6)	n.r.	29.8%	5‑year: 73.5%	n.r.	5‑year: 91%
						T_min_^b^ = 39.1 (95%CI 37.6–40.6)					
						T_max_^b^ = 40.6 (39.5–41.6)					
						T_20_^b^ = 40.2 (95%CI 39.3–41.2)					
						T_50_^b^ = 39.9 (39.1–40.7)					
						T_90_^b^ = 39.3 (95%CI 38.5–40.1)					
Lee et al. [27]	40/20	400 5‑FU or 825 capecitabine	Capacitive	8 (8–9)	n.r.	n.r.	58 (6–85)	15%	5‑year: 94%	5‑year: 77.1%	5‑year: 96.4%
Ott et al. [7]	45–50.4/25–28	250 5‑FU or 1650 capecitabine + 50 oxaliplatin	Radiative	10 (1–11)	6.4^b^ ± 5.2	n.r.	34 (0–81)	19%	5‑year: 75% LARC 82% LRRC 46%	5‑year: LARC 57% LRRC 37%	5‑year: LARC 77% LRRC 49%
Gani et al. [8]	50.4/28	1000 5‑FU	Radiative	n.r.	4.5^b^ (IQR: 2.2–8.2)	T_90_ ^b^= 39.5 (IQR 39.1–39.9)	54	14%	3‑year: 94%	3‑year: 81%	3‑year: 96%
Shoji et al. [28]	50/25	1700 capecitabine	Capacitive	n.r.	n.r.	n.r.	n.r.	20.4%	n.r.	n.r.	n.r.
Gani et al. [24]	50.4/28	1000 5‑FU	Radiative	4 (1–9)	1.1^c^ (0–9.2)	T_90_^c^ = 39.3 (37.1–40.6)	58.8	61% reported in [11]	5‑year: 88%	5‑year: 77%	5‑year: 98%
Kato et al. [29]	50/25	250 5‑FU + 25 levofolinate calcium	Capacitive	4 (2–5)	n.r.	n.r.	32 (4–102)	29.2%	n.r.	n.r.	n.r.
Barsukov et al. [30]	40/10	650 capecitabine + 50 oxaliplatin	Capacitive	4^a^	n.r.	n.r.	24.9	11%	2‑year: 91%	2‑year: 83%	2‑year: 86.4%
Kang et al. [25]	Group A 39.6/22	425 5‑FU + 20 mg/kg leucovorin + 10 mitomycin C	Capacitive	Group A 7 (1–11)	n.r.	Group A T_max_^b^ = 40 ± 0.9	Group A 102 (2–173)	Group A 15.8%	Group A 5‑year: 73.4%	Group A 5‑year: 78.8%	Group A 5‑year: 94.8%
	Group B 45/25			Group B 9 (1–11)		Group B T_max_^b^ = 39.5 ± 0.7	Group B 53 (3–93)	Group B 13.3%	Group B 5‑year: 74.4%	Group B 5‑year: 72.1%	Group B 5‑year: 93.1%
Maluta et al. [13]	50/25	200 5‑FU + 46 oxaliplatin	Radiative	5	n.r.	T_90_^b^ = 40.8 (95%CI 40.6–41)	51 (24–83)	23.6%	5‑year: 86.5%	5‑year: 74.5%	5‑year: 94.6%
						T_max_^b^ = 41.6 (95%CI 41.4–41.8)					
Rau et al. [31]	45–50.4/25–28	50 mg leucovorin + 300 5‑FU	Radiative	5	CEM T_90_ ≥ 40.5 °C: 120–150	n.r.	21	14%	3‑year: 86%	n.r.	n.r.

*n.r.* not reported, *RT* radiotherapy, *HT* deep regional hyperthermia, *FU* follow-up, *pCR* pathological complete response, *OS* overall survival, *LRFS* local recurrence-free survival, *5-FU *5‑fluorouracil, *CEM* cumulative minutes at, *95%CI* 95% confidence interval, *IQR* interquartile range

^a^prescribed

^b^mean

^c^median

Table 2 shows that patients in six studies were treated with the radiative radiofrequency BSD 2000 HT device (Pyrexar Medical, Salt Lake City, USA) [7, 12, 13, 24, 31, 32] and in six studies using various capacitive radiofrequency HT devices [25–30].

### Primary outcome: pCR rate

**Fig. 2:**
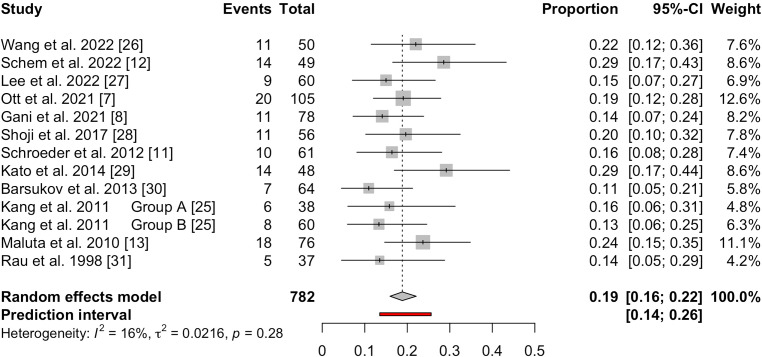
Forest plot of pCR rate for LARC and LRRC patients using a random-effects model. Individual pCR rate for each study and the pooled weighted estimate are shown with 95%CI. The *vertical dotted line* represents the pooled weighted estimate. Each square represents an indvidual study and the horizontal lines indicate the 95% confidence intervals. The overall pooled estimate is represented by the diamond at the bottom.

All included studies reported the pCR rate after neoadjuvant CRT combined with HT [7, 12, 13, 24–31]. When the data from all studies were pooled, the pCR rate of 782 patients was 19% (95%CI: 16–22%) and the prediction interval was 14–26% (Fig. 2). There was moderate heterogeneity among the studies (*I*^*2*^ = 16%). Influence analysis revealed that none of the studies were influential regarding the overall effect (supplementary Figure S1). The pooled pCR rate of 760 LARC patients was 19% (95%CI: 16–23%, *I*^*2*^ = 27%) and the prediction interval was 12–28% (supplementary Figure S2). The influence analysis showed that none of the studies contributed to overall heterogeneity of the pooled pCR rate in LARC patients. Out of 12 clinical studies, only two clinical trials included LRRC patients [7, 12] and the pooled pCR rate of 22 LRRC patients was 16% (95%CI: 6–38%, *I*^*2*^ = 0%). Based on influence analysis, the HyRec trial was revealed to be influential regarding the overall effect of the pooled pCR rate of LRRC patients.

There were in total 7 out of 12 included clinical studies that reported ypT or ypN staging after surgery [8, 11, 13, 25, 27, 29, 31]. The pooled ypT downstaging rate for 453 LARC patients who underwent surgery was 54% (95%CI: 49–60%), as shown in supplementary Figure S3. None of the studies were influential regarding the pooled overall effect. The data from eight clinical studies [8, 11, 13, 25, 27, 29–31] were significantly heterogenous (*I*^*2*^ = 71%) when the ypN0 rate was pooled. The influence analysis indicated that the study by Kato et al. [29] was particularly influential regarding the overall effect. This study was excluded, and after re-pooling the data, the ypN0 rate among 453 patients was 71% (95%CI: 66–77%).

The number of HT sessions given per week differed among the 12 studies. In the six studies where HT was given twice weekly [7, 8, 11, 25–27], the pooled pCR rate of 452 patients was 17% (95%CI: 14–21%). The percentage of variation across these six studies due to heterogeneity was small (*I*^*2*^ = 0%). In the six studies where HT was given once a week (maximum of five HT sessions) combined with CRT, the pCR rate of 326 patients was 21% (95%CI: 16–28%). There was moderate heterogeneity among the six (*I*^*2*^ = 45%) studies and the 95%CI was very large. The influence analysis revealed that the heterogeneity of the pooled data would be reduced to 2% by omitting the study of Barsukov et al. [30]. The calculated pooled pCR rate after removal of this study was 24% (95%CI: 19–30).

Temperature metrics and thermal dose CEM43 were not consistently reported across the 12 studies. Therefore, temperature data could not be pooled and analyzed for an association with clinical outcome. Four studies reported the mean or median estimated temperature achieved in 90% of the measured points (T_90_) [8, 12, 13, 24]. Four studies reported thermal dose CEM43 [7, 8, 24, 26] and one study reported cumulative minutes at T_90_ ≥ 40.5 °C in lieu of CEM43 [31]. The performed univariate meta-regressions revealed that there is no relationship of pCR rate with total radiation dose, chemotherapy regimen, HT technique, or total HT sessions.

### Secondary outcomes: OS, DFS, LRFS, and acute toxicity

The 5‑year OS rates were reported in seven studies ([7, 12, 13, 24–27]; Table 2). The pooled 5‑year OS rate among 498 patients was 85% (95%CI: 79–89%; supplementary Figure S4). There was high and significant heterogeneity among the studies (*I*^*2*^ = 59%, *p* = 0.02), and the 95%CI (95%CI: 79–89%) was large. The influence analysis showed that removal of the data from the HyRec trial, which contained data from both LARC and LRRC patients, might affect the analysis (supplementary Figure S5). After exclusion of the HyRec trial, the pooled 5‑year OS rate for 433 LARC patients from six studies [7, 13, 24–27] was 87% (95%CI: 83–90%), as shown in supplementary Figure S6. There was low heterogeneity among the studies (*I*^*2*^ = 0%). Only two studies reported the 3‑year OS rate [8, 31], and the data were thus not pooled.

**Fig. 3:**
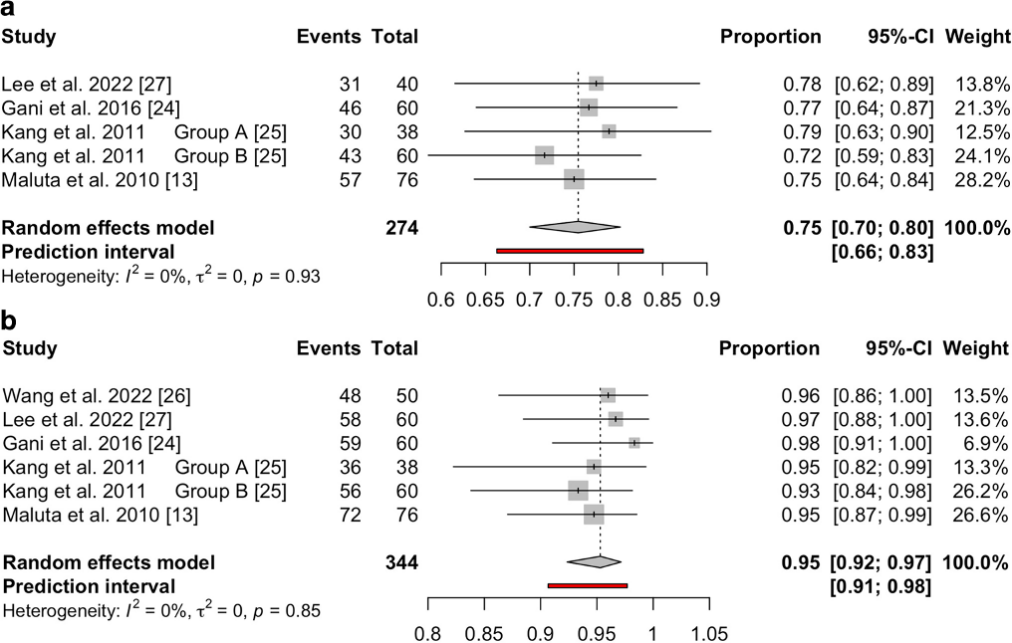
Forest plot of 5‑year (**a**) DFS and (**b**) LRFS for rectal cancer patients using a random-effects model. Individual (**a**) DFS and (**b**) LRFS rates for each study and the pooled weighted estimate are shown with 95%CI. The *vertical dotted line* represents the pooled weighted estimate. Each square represents an indvidual study and the horizontal lines indicate the 95% confidence intervals. The overall pooled estimate is represented by the diamond at the bottom.

The 5‑year DFS rate of only LARC patients was pooled because one of the two studies that included LRRC patients [12] did not report DFS as a clinical endpoint. Instead, 5‑year relapse-free survival and cancer-specific survival rates were evaluated. In total, six studies [7, 13, 24–27] reporting the 5‑year DFS rate were used for pooled analysis (Table 2). Pooled 5‑year DFS among 413 LARC patients was 71% (95%CI: 64–77%), with high and significant heterogeneity among the studies (*I*^*2*^ = 53%, *p* = 0.05). The influence analysis showed that the HyRec trial and the retrospective study from Wang et al. [26] influenced the overall pooled 5‑year DFS rate. After excluding these two studies [7, 26], the pooled 5‑year DFS rate among 274 LARC patients was 75% (95%CI: 70–80%) and the prediction interval was 66–83%, as shown in Fig. 3a. There was low and nonsignificant heterogeneity among the studies (*I*^*2*^ = 0%, *p* = 0.93).

The 5‑year LRFS rate of 498 LARC and LRRC patients in seven clinical studies [7, 12, 13, 24–27] was 94% (95%CI: 91–95%, *I*^*2*^ = 0%). The influence analysis showed that the HyRec trial was influential, and it was therefore excluded. The pooled 5‑year LRFS rate of 393 patients (6 LRRC, 387 LARC) in six studies [12, 13, 24–27], excluding the data of HyRec trial, was 95% (95%CI: 92–97%), as shown in supplementary Figure S7. The influence analysis showed that none of the remaining studies were influential regarding the overall pooled outcome. The pooled 5‑year LRFS rate of 344 LARC patients in five clinical studies [13, 24–27] was 95% (95%CI: 92–97%), with a low heterogeneity (*I*^*2*^ = 0%, *p* = 0.85), as shown in Fig. 3b.

Reporting of toxicity was inconsistent across the studies; therefore, toxicity data were not pooled. Toxicity symptoms greater than grade 3 are summarized in supplementary Table S4.

### Publication bias

Of the 12 included studies, 9 were evaluated with a total NOS score of 6 and, three studies with a respective score of 8 (supplementary Table S5; [7, 8, 12, 13, 24–31]). Two studies reported comparative clinical data from unexposed groups [24, 25]. In 12 studies, the population was representative or somewhat representative of the average population. Publication bias was evaluated using the funnel plot method with Egger’s regression test. No statistically significant publication bias was observed in the funnel plots and Egger’s regression test (*p* = 0597; supplementary Figure S8). In addition, a summary of the GRADE assessment for the certainty of evidence is provided in supplementary Table S6.

## Discussion

This is the first systematic review and meta-analysis performed to assess the effect of HT when used in combination with neoadjuvant CRT on the pCR rate in rectal cancer patients with LARC and LRRC. We included 12 studies in the meta-analysis, encompassing 778 patients. The pooled pCR rate was 19% (95%CI: 16–22%). The pooled pCR rate in LARC was 19% (95%CI: 16–23%), 5‑year OS was 87% (95%CI: 83–90%), 5‑year DFS was 75% (95%CI: 70–80%), and 5‑year LRFS rate was 95% (95%CI: 92–97%). Furthermore, very moderate rates of high-grade toxicity symptoms were reported in the clinical studies that applied different chemotherapeutic drugs and regimens in combination with radiation therapy and HT.

The radiosensitizing effects induced by temperature at 40–43 °C depend on the temperature achieved during the HT treatment sessions. Temperature parameters recorded during HT sessions usually include thermal dose CEM43 or the maximum temperature achieved during the treatment. In patients with rectal cancer treated with CRT + HT, Schem et al. found a temperature-dependent relapse-free survival rate, which reached 66.7% for patients with recordings above a median T_50_ of 39.9 °C versus 31.3% in the lower temperature group (*p* = 0.047) [12]. Moreover, Gani et al. reported that LARC patients treated with higher CEM43 had improved tumor regression [8]. Additionally, the analysis performed in 37 patients enrolled in a phase II clinical trial study showed that pCR was associated with T_90_ and CEM43 at T_90_ (CEM43T_90_) [31]. The temperature dependency of treatment efficacy in several studies underscores the fact that HT is an effective treatment option. However, the inconsistent reporting of HT temperature parameters unfortunately made it impossible to pool the data and correlate them with outcome in this study.

Only one study, which used a capacitive HT device, documented the temperature according to the European Society for Hyperthermic Oncology (ESHO) quality assurance (QA) guidelines [25], which recommend using tumor thermometry to guide treatment delivery [33]. In this study, Kang et al. reported that the mean maximum tumor temperature (T_max_) was 40 ± 0.9 °C in group A and 39.5 ± 0.7 °C in group B ([25]; Table 2). These temperatures were 1.6 and 2.1 °C lower, respectively, compared to those reported in Maluta et al.’s phase II trial, where patients received treatment with a radiative HT device [13], and 0.6 and 1.1 °C lower than those in Schem et al.’s phase II trial, where a radiative HT device was also used [12]. The observed differences of more than 0.5 °C could be attributed to the difficulties in achieving deep tumor heating with capacitive devices, despite the favorable eccentric location of rectal tumors [34], particularly if combined with the use of an overlay bolus [35] and vigorous bolus cooling [36]. Capacitive HT faces challenges with overheating at the edges of electrodes and in the subcutaneous fat layer. Better heating results are seen in patients with less than 1.5 cm of subcutaneous fat [37], and vigorous cooling can help to achieve the target temperature in patients with up to 3 cm of fat [36].

The preoperative radiotherapy schedule of LARC patients is either (i) CRT to a total radiotherapy dose of 50.4 Gy/28 fractions with concurrent chemotherapy without using a watch-and-wait strategy or a total dose of 50–56 Gy combined with concurrent chemotherapy following a watch-and-wait strategy or (ii) short radiotherapy treatment course with a total dose of 25 Gy/5 fractions [1–3]. The local efficacy of CRT was demonstrated in a landmark phase III trial (CAO/ARO/AIO-94) [9]. In this trial, the pCR rate of 8% and 5‑year DFS of 68% are lower than our pooled results (pCR: 19% and 5‑year DFS: 75%). Subsequently, the randomized CAO/ARO/AIO-04 trial investigated the addition of oxaliplatin to concurrent and adjuvant 5‑FU-based chemotherapy, which resulted in a significantly improved DFS of patients with clinically staged cT3–4 or cN1–2 rectal cancer compared with the 5‑FU-based chemotherapy combined-modality regimen [38]. Based on these data, oxaliplatin was added to the standard of care in Germany. The CAO/ARO/AIO-04’s investigational and control arms reported pCR rates of 17% and 13%, respectively [38], with the control arm incorporating a similar CRT regimen, but without adding HT. Furthermore, a systematic review and meta-analysis, which included eight randomized clinical trials conducted in Germany, the USA, France, Korea, Italy, and China, was performed to evaluate whether the addition of oxaliplatin to neoadjuvant CRT and adjuvant chemotherapy benefits patients with LARC [39]. In this meta-analysis, it was found that neoadjuvant CRT based on 5‑FU plus oxaliplatin achieved a higher pCR rate (odds ratio [OR] = 1.29 [95%CI: 1.12–1.49]) and a higher 3‑year DFS rate (OR = 1.15 [95%CI: 0.93–1.42]) compared to CRT based on 5‑FU alone, but no difference was found between the two regimens in terms of 3‑year OS or 5‑year DFS rates. A more recent literature review also suggested that adding oxaliplatin can enhance the response to neoadjuvant CRT; however, these effects do not translate into improved long-term outcomes including OS and DFS [40].

Several randomized studies have recently reported the efficacy of intensified TNT regimens combining either CRT or short-course radiotherapy with intensified chemotherapy. Patients enrolled in the investigational arm of the RAPIDO TNT trial received short-course radiotherapy followed by six cycles of capecitabine/oxaliplatin and nine cycles FOLFOX followed by surgery [2]. The intensified chemotherapy regimen significantly improved the 3‑year disease-related treatment failure (23.7% vs. 30.4%) and pCR rate (28% vs. 14%) in comparison to the standard-of-care group treated with preoperative CRT alone [2]. Similarly, in the PRODIGE-23 randomized clinical trial, a TNT regimen of six cycles FOLFIRINOX followed by CRT and, after 7 weeks, surgery followed by an additional six cycles of FOLFOX also significantly improved 3‑year DFS (75.7% vs. 68.5%) and pCR (28% vs. 12%) if compared to the standard arm of neoadjuvant CRT alone but failed to improve the local relapse rate [10]. Importantly, part of the TNT strategy is to increase the time between radiotherapy and surgery to allow more time to achieve complete tumor regression. In the control arms of the RAPIDO and PRODIGE-23 studies, which are comparable to our patient cohort with regard to neoadjuvant treatment regimen and time to surgery, the pCR rates were 12% and 14% [2, 10]. No TNT study so far has integrated HT. Recently, the randomized phase II OPRA trial showed that LARC patients who were treated with either NCT-CRT or CRT-CNCT followed by total mesorectal excision or watch and wait had the same 3‑year DFS rate regardless of the treatment arm (76% [95%CI 69–84] vs. 76% [95%CI 69–83]; *p* = 0.98) [5]. Similarly, the pathological complete response in LARC patients who underwent surgery in each treatment group of the OPRA trial, NCT-CRT and CRT-CNCT, did not differ (6/158 [8%] vs. 6/166 [9%]) [5]. In the group of patients who underwent a watch-and-wait strategy and who developed local recurrence, tumor regrowth occurred mostly in the first 2 years [41]. Furthermore, the OPERA trial demonstrated that an organ preservation rate of up to 81% can be achieved in patients with early rectal cancer who receive a high radiation dose with a brachytherapy boost following neoadjuvant chemoradiotherapy with 45 Gy, which was significantly higher compared to patients who received a radiation dose boost dose of 9 Gy (*p* = 0.02) [6]. Assessing the effect of radiation dose on our pooled data is challenging due to the variability in prescribed total doses and fractionation schemes among the included studies (Table 2). Among these studies, the Norwegian single-arm phase II trial administered the highest dose of neoadjuvant chemoradiotherapy (54–56 Gy) combined with hyperthermia, followed by surgery after 6–8 weeks. This study reported a pCR rate of 29.8%, which was the highest compared to other prospective and retrospective clinical studies included in the analysis [12]. However, this should be interpreted with caution due to potential differences in patient characteristics, in the temperatures achieved in the target volume during hyperthermia sessions, and in the chemotherapy regimens among the patients in these studies.

The ongoing CAO/ARO/AIO-16 pilot phase II trial was designed to investigate organ preservation by CRT followed by consolidation chemotherapy, and HT could additionally be combined with CRT at the centers in Tübingen und Erlangen. Patients received a total radiation dose of 50.4 Gy and concurrent 5‑FU and oxaliplatin in combination with HT (only two clinical centers) followed by consolidation chemotherapy (folinic acid, oxaliplatin, 5‑FU). Surgery was omitted in the event of clinical complete response (cCR) or a near complete response. The preliminary analysis of this study has recently been presented and showed highly promising complete response rates (pCR rate in the case of surgery or cCR in the case that surgery was not performed) of 40% [32].

The relatively high pooled pCR rate that includes patients with LRRC corresponds to findings of previous studies showing that LRRC patients can also substantially benefit from the combination of neoadjuvant CRT and HT [7, 12]. One biological explanation for this effect is the specific tumor biology of recurrent cancers, where HT does not only induce direct cytotoxicity of tumor cells [42] but is also able to radiosensitize pre-irradiated and treatment-resistant hypoxic tumors by increasing tumor blood flow and thus the perfused fraction of a tumor in a temperature- and time-dependent manner [43, 44].

The current study has several limitations, which are mainly attributed to the design of the included studies. Firstly, all studies had several methodological quality issues, such as different selection biases, inclusion criteria, and treatment variations as well as reporting of clinical outcomes. Furthermore, the primary endpoint for this meta-analysis was pCR, which depends not only on the intensity of neoadjuvant CRT but also on the time interval between CRT and surgery as well as on the technique of pathological evaluation and reporting of specimens. Another limitation of this study is the low number of patients included in the studies, despite exclusion of the pilot studies [45, 46]. The current analysis was not a specific individual patient data analysis; thus, it was not possible to perform analyses according to specific cancer stage categories or risk factors. The small size and high variability of parameters limited our analysis to creating a meta-regression model to evaluate the relationship of tumor and treatment parameters with clinical outcomes. Heterogeneity from the use of different chemotherapy treatment regimens and radiation doses also limits the interpretation of the data. Furthermore, the data from the retrospective studies with comparative arms were not pooled to estimate the odds ratio because there was no evaluation to assess the balance of specific covariates between treatment arms (e.g., propensity score-matched analysis), nor were there sample size calculations to determine the minimum sample size required to detect differences between treatment arms [11, 25, 26]. Regarding statistical analysis, one of the main limitations was that the median survival could not be used for survival analysis, because the median survival time was not reached in 11 out of 12 included studies. Lastly, a potential limitation of our study is the selection strategy to avoid patient overlap in clinical studies, as we included only the largest study when overlapping patients were identified, while excluding studies that included overlapping patients from the same research group. Including the larger study in the case of overlapping study periods may have led to the exclusion of more non-overlapping subjects than necessary for the meta-analysis, resulting in a smaller sample size. However, we found that this was a transparent and reliable approach to avoiding overlap, which may have introduced even greater bias. The risk of bias is difficult to accurately define in meta-analyses of observational studies [47]. However, prospective and retrospective studies provide valuable supplementary information regarding safety and long-term outcomes of interventions. Their results might be more directly applicable to a general population, as they are conducted in a real-world setting in contrast to randomized clinical trials, which usually involve very restricted populations treated with highly standardized care.

Certainly, our results should be interpreted with caution because no evidence from a randomized study investigating the effect of HT in combination with preoperative CRT in rectal cancer patients is currently available. Nevertheless, the results of our meta-analysis support the conduct of a randomized trial to inform physicians treating rectal cancer regarding the option of HT for selected patients, e.g., those with very advanced or recurrent tumors.

## Conclusion

The present study summarizes the current clinical evidence in rectal cancer patients and shows that HT in combination with neoadjuvant CRT results in high pCR rates in comparison to comparable contemporary patient cohorts. A randomized study investigating this strategy should be conducted. In addition, this strategy should be explored in the setting of TNT and watch-and-wait treatment concepts.

## Supplementary Information

Supplementary tables S1–S6 and supplementary figures S1–S8

## Data Availability

All the data generated or analyzed during this study are included in the published studies. Research data are stored in an institutional repository and will be shared upon reasonable request to the corresponding author.
