# Supplementary material for: Neoadjuvant chemoradiotherapy in combination with deep regional hyperthermia followed by surgery for rectal cancer: a systematic review and meta-analysis
Source: Strahlenther Onkol. 2024 Oct 17;201(2):151–62. doi: 10.1007/s00066-024-02312-9 (PMC11754347; doi:10.1007/s00066-024-02312-9)
Supplement: Supplementary file 1 — Supplementary tables S1–S6 and supplementary figures S1–S8 [file 66_2024_2312_MOESM1_ESM.docx]

**Supplementary material**

**Neoadjuvant radiochemotherapy in combination with regional deep hyperthermia followed by**

**surgery for rectal cancer: a systematic review and meta-analysis**

| **Section and topic** | **Item No** | **Checklist item** | | **Inclusion**  **(Section or page number)** |
| --- | --- | --- | --- | --- |
| **ADMINISTRATIVE INFORMATION** | | |  |  |
| Title:   - Identification - Update | 1a  1b | Identify the report as a protocol of a systematic review  If the protocol is for an update of a previous systematic review, identify as such | | Title  n/a |
| Registration | 2 | If registered, provide the name of the registry (such as PROSPERO) and registration number | | n/a |
| Authors:   - Contact - Contributions | 3a  3b | Provide name, institutional affiliation, e-mail address of all protocol authors; provide physical mailing address of corresponding author  Describe contributions of protocol authors and identify the guarantor of the review | | Title page  Page 14 |
| Amendments | 4 | If the protocol represents an amendment of a previously completed or published protocol, identify as such and list changes; otherwise, state plan for documenting important protocol amendments | | n/a |
| Support:   - Sources - Sponsor - Role of sponsor or funder | 5a  5b  5c | Indicate sources of financial or other support for the review  Provide name for the review funder and/or sponsor  Describe roles of funder(s), sponsor(s), and/or institution(s), if any, in developing the protocol | | No funding  n/a  n/a |
| **INTRODUCTION** | | |  |  |
| Rationale | 6 | Describe the rationale for the review in the context of what is already known | | Page 5 |
| Objectives | 7 | Provide an explicit statement of the question(s) the review will address with reference to participants, interventions, comparators, and outcomes (PICO) | | Page 5 |
| **METHODS** | | |  |  |
| Eligibility criteria | 8 | Specify the study characteristics (such as PICO, study design, setting, time frame) and report characteristics (such as years considered, language, publication status) to be used as criteria for eligibility for the review | | Page 6 |
| Information sources | 9 | Describe all intended information sources (such as electronic databases, contact with study authors, trial registers or other grey literature sources) with planned dates of coverage | | Page 6 |
| Search strategy | 10 | Present draft of search strategy to be used for at least one electronic database, including planned limits, such that it could be repeated | | Supplemental Table 2 |
| Study records:   - Data management - Selection process      - Data collection process   Data items | 11a  11b  11c  12 | Describe the mechanism(s) that will be used to manage records and data throughout the review  State the process that will be used for selecting studies (such as two independent reviewers) through each phase of the review (that is, screening, eligibility and inclusion in meta-analysis)  Describe planned method of extracting data from reports (such as piloting forms, done independently, in duplicate), any processes for obtaining and confirming data from investigators  List and define all variables for which data will be sought (such as PICO items, funding sources), any pre-planned data assumptions and simplifications | | Page 6-7  Page 6-7  Page 7  Page 7 |
| Outcomes and prioritization | 13 | List and define all outcomes for which data will be sought, including prioritization of main and additional outcomes, with rationale | | Page 7 |
| Risk of bias in individual studies | 14 | Describe anticipated methods for assessing risk of bias of individual studies, including whether this will be done at the outcome or study level, or both; state how this information will be used in data synthesis | | Page 7-8 |
| Data synthesis | 15a  15b  15c  15d | Describe criteria under which study data will be quantitatively synthesized  If data are appropriate for quantitative synthesis, describe planned summary measures, methods of handling data and methods of combining data from studies, including any planned exploration of consistency (  such as I2, Kendall’s τ)  Describe any proposed additional analyses (such as sensitivity or subgroup analyses, meta-regression)  If quantitative synthesis is not appropriate, describe the type of summary planned | | Page 7-8  Page 7-8  n/a |
| Meta-bias(es) | 16 | Specify any planned assessment of meta-bias(es) (such as publication bias across studies, selective reporting within studies) | | Page 7-8 |
| Confidence in cumulative evidence | 17 | Describe how the strength of the body of evidence will be assessed (such as GRADE) | | GRADE |

**Table S1.** Preferred Reporting Items for Systematic review and Meta-Analysis (PRISMA) protocols checklist: recommended items to address in a systematic review protocol. *From Shamseer, L., Moher, D., Clarke, M., Ghersi, D., Liberati, A., Petticrew, M., Shekelle, P., Stewart, L. A., & PRISMA-P Group (2015). Preferred reporting items for systematic review and meta-analysis protocols (PRISMA-P) 2015: elaboration and explanation. BMJ (Clinical research ed.), 350, g7647. https://doi.org/10.1136/bmj.g7647*

| **Database: PubMed**  Data searched: till 10 November 2022  Records retrieved: 61  PubMed database has been searched independently by two investigators (AA and SB) using the following terms:  radiochemotherapy [MeSH] OR chemoradiotherapy [MeSH] AND hyperthermia [MeSh] AND rectal cancer [MeSH] OR rectum cancer [MeSH] |
| --- |
| **Database: Scopus**  Data searched: 06 December 2022  Records retrieved: 77  Similar literature search to PubMed, Scopus database has been searched independently by two investigators (AA and SB) using the following terms   \| #1 \| (radiochemotherapy AND hyperthermia AND rectal cancer) \| \| --- \| --- \| \| #2 \| (radiochemotherapy AND hyperthermia AND rectum cancer) \| \| #3 \| (chemoradiotherapy AND hyperthermia AND rectal cancer) \| \| #4 \| (chemoradiotherapy AND hyperthermia AND rectum cancer) \| |
| **Database: Mendeley**  Data searched: 21 April 2023  Records retrieved: 81  S1 chemoradiotherapy OR radiochemotherapy AND hyperthermia AND rectal cancer OR rectum cancer |

**Table S2**. Search strategy.

| First Author | n | Surgery after neoadjuvant treatment (weeks) | Surgical interventions |
| --- | --- | --- | --- |
| Wang | 50 | 10.154 ± 2.36^†^ | Miles: 18 (36.0%)  Dixon: 27 (54.0%)  Hartmann: 5 (10.0%) |
| Schem | 49 | 12 (9.8–30.85) ^‡^ | No details are provided |
| Lee | 60 | 7 (6–10) ^‡^ | Low anterior resection: 50 (83.3%)  Abdominoperineal resection: 4 (6.7%)  Hartmann’s procedure: 3 (5.0%)  Total exenteration: 1 (1.7%)  Transanal excision: 1 (1.7%)  Intersphincteric resection: 1 (1.7%) |
| Ott | 105 | 4–8 * | No details are provided |
| Gani | 78 | 6.7 (IQR 5.7–7.4) ^‡^ | Low anterior resection: 52 (67%)  Abdominoperineal resection: 26 (33%) |
| Shoji | 81 | 15(8–42) ^‡^ | APR Miles: 12 (14.8%)  ISR: 7 (8.6%)  sLAR: 15 (18.5%)  LAR: 13 (16.0%)  Local incision: 6 (7.4%)  Pelvic: 1 (1.2%)  No resection: 2 (2.5%)  No surgery: 25 (30.9%) |
| Schroeder | 61 | 4–6* | Sphincter sparing surgery: 40 (66%) |
| Kato | 48 | 5–34^‡^ | TME with pelvic autonomic nerve preservation: 42 (87.5%) |
| Barsukov | 64 | 6–8* | Radical tumor resection: 59 (92.2%);  Sphincter sparing surgery: 36 (61%); |
| Kang | 98 | 4–6* | TME: routinely performed |
| Maluta | 76 | 4–6* | TME: 56 (73.7%)  Miles: 20 (26.3%) |
| Rau | 37 | 4–6* | Sphincter preserving: 19 (51%)  Anterior resection: 3 (8%) |

**Table S3.** Surgical interventions reported in included studies. ^†^: mean value ± standard deviation; ^‡^: median value (range); *: prescribed according to treatment standard or protocol; TME: Total mesorectal excision.


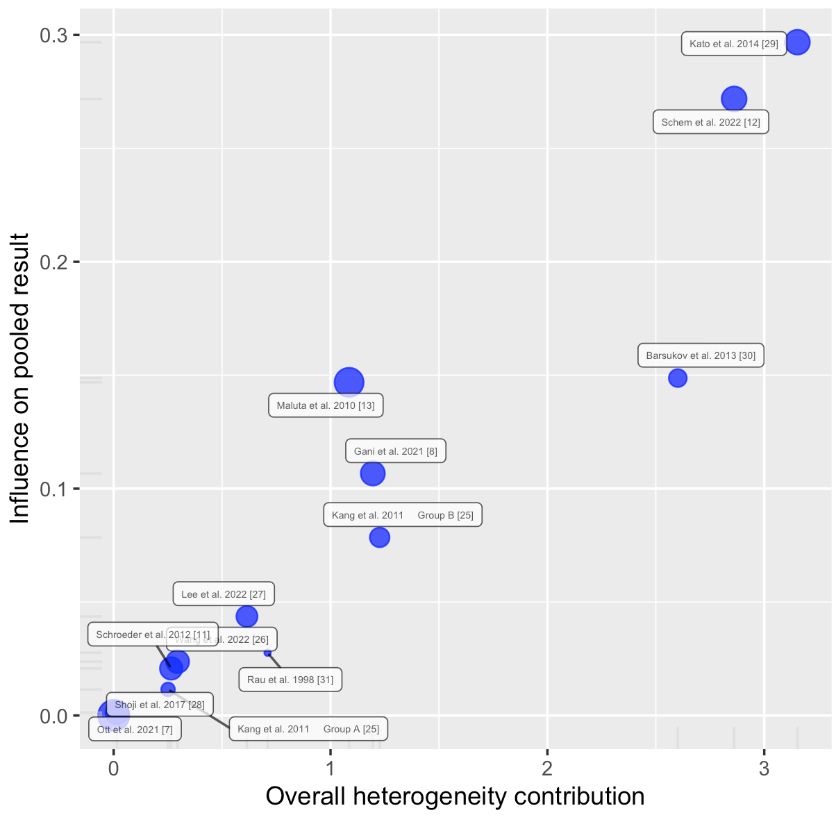


**Figure S1.** Influence analyses of studies reporting on pathological complete response (pCR) in rectal cancer patients.


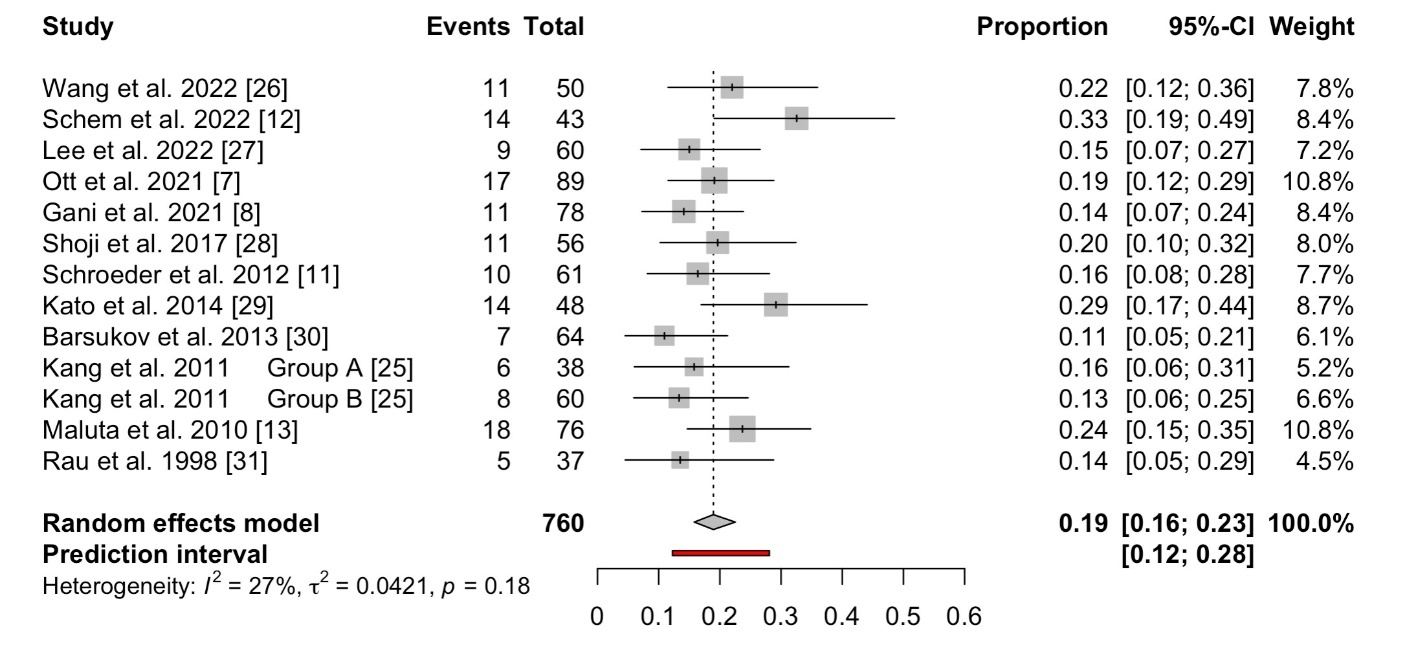


**Figure S2.** Forest plot of pCR rate for LARC patients using a random-effects model. Individual pCR rate for each study and the pooled weighted estimate are shown with 95%CI. Vertical dotted line represents the pooled weighted estimate.


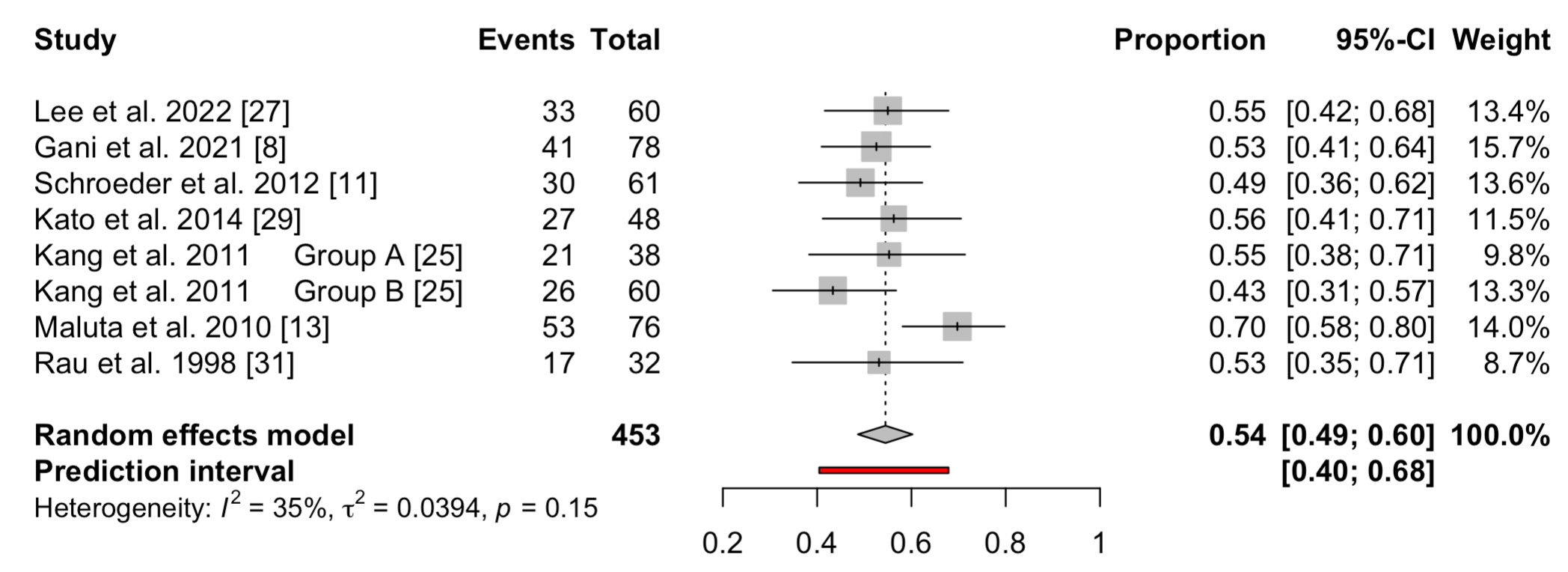


**Figure S3.** Forest plot of ypT rate for LARC patients who underwent surgery after neoadjuvant chemoradiotherapy using a random-effects model.


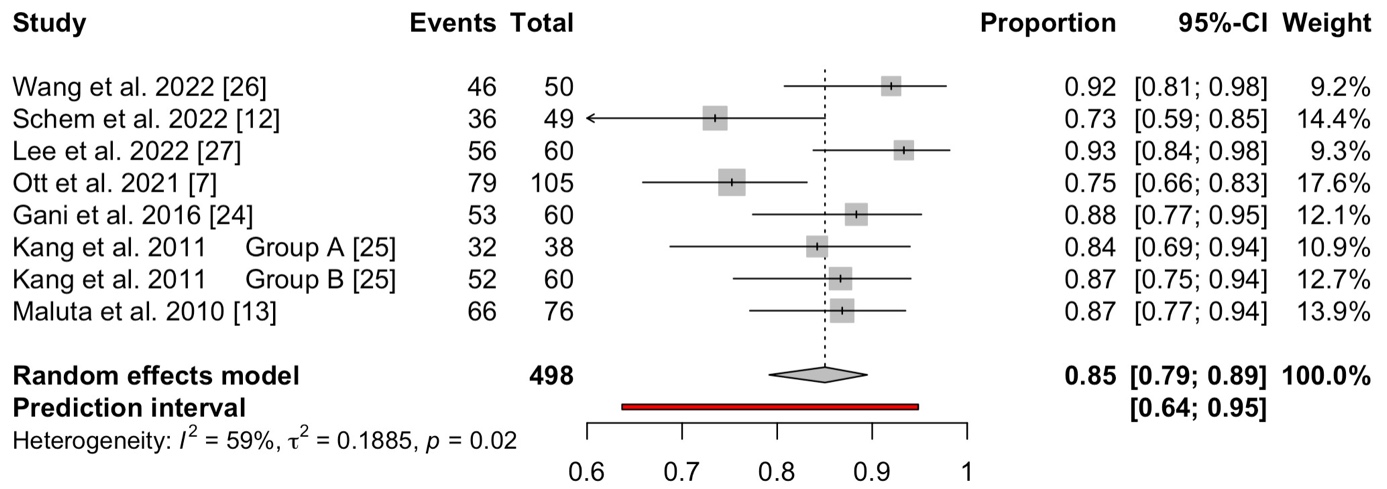


**Figure S4**. Forest plot of 5-year overall survival (OS) rate for LARC and LRRC patients using a random-effects model. Individual 5-year OS rate for each study and the pooled weighted estimate are shown with 95% confidence interval (CI). Vertical dotted line represents the pooled weighted estimate.


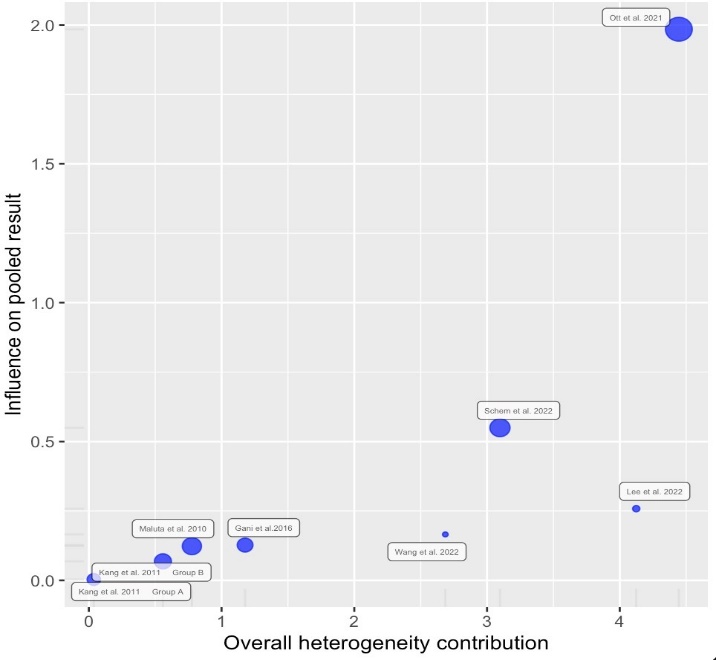

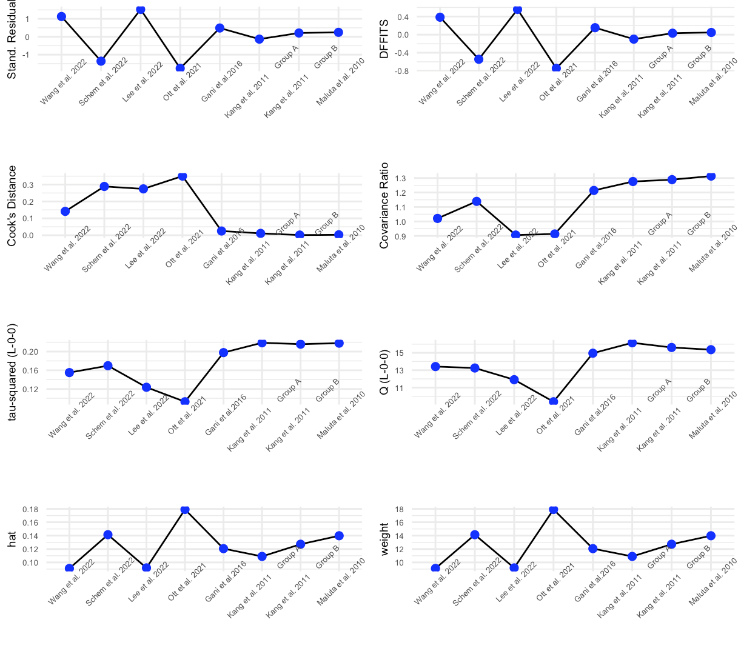


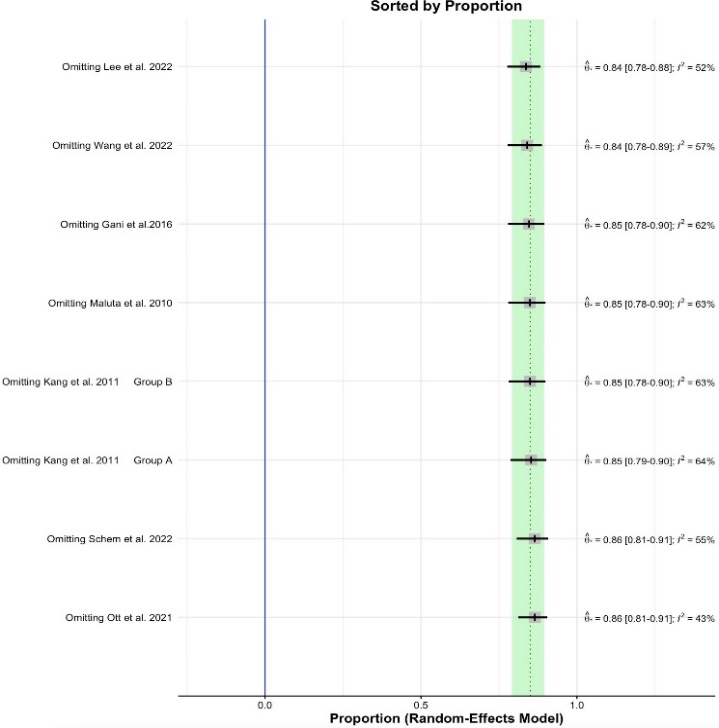

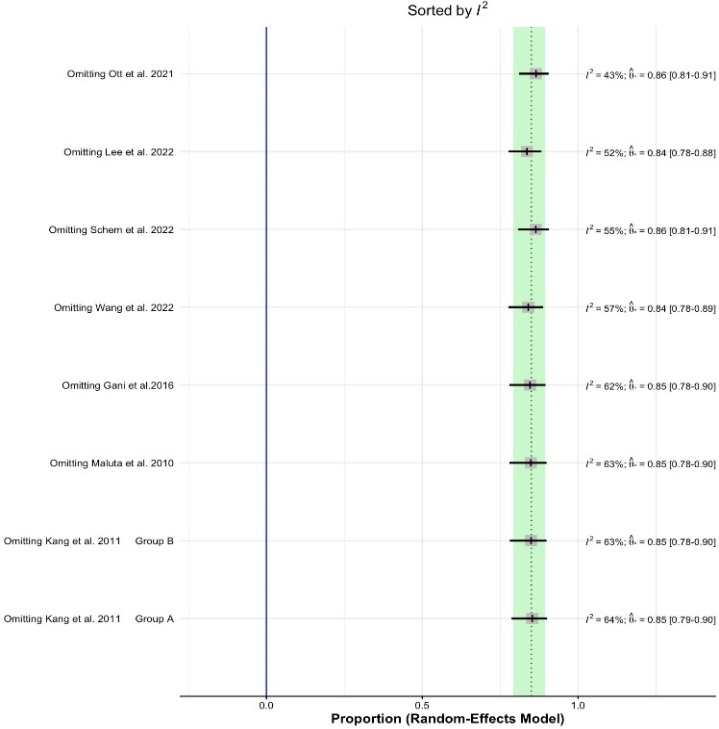


**Figure S5.** Influence analyses studies reporting 5-year OS rate.


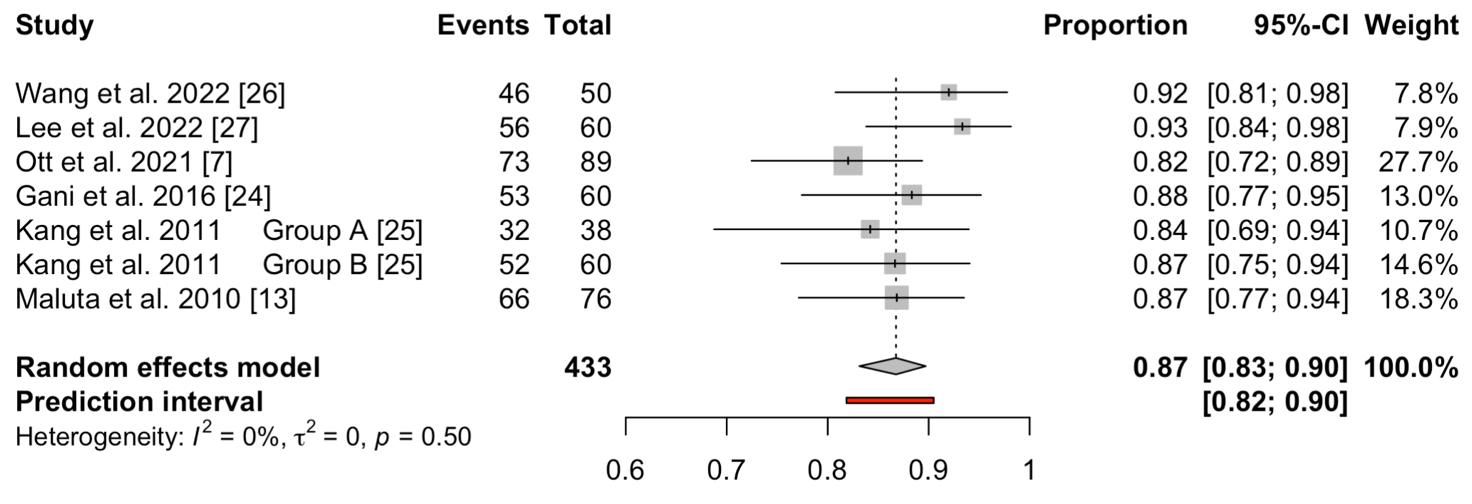


**Figure S6.** Forest plot of 5-year OS for LARC patients using a random-effects model. Individual OS rate for each study and the pooled weighted estimate are shown with 95%CI. Vertical dotted line represents the pooled weighted estimate


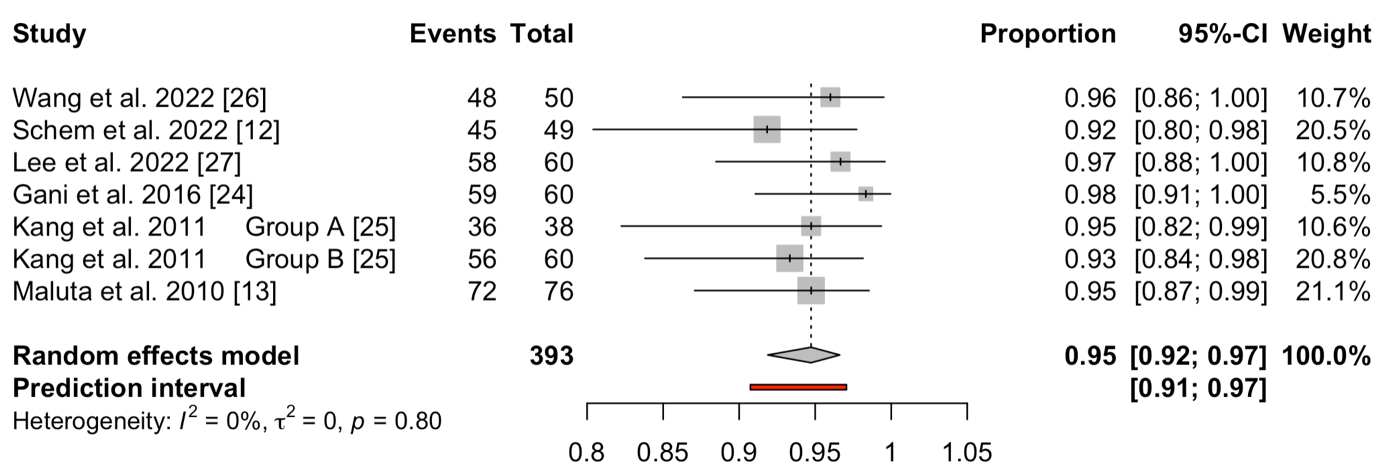


**Figure S7.** Forest plot of 5-year LRFS for LARC patients using a random-effects model. Individual LRFS rate for each study and the pooled weighted estimate are shown with 95%CI. Vertical dotted line represents the pooled weighted estimate

| Study | n | Grade 3 (%) | Grade 4-5 (%) |
| --- | --- | --- | --- |
| Schem et al. 2022 | 49 | 19 | 2 |
| Lee et al. 2022 | 60 | 0 | 0 |
| Ott et al. 2021 | 105 | 29 | 0 |
| Shoji et al. 2017 | 81 | 7 | 0 |
| Kato et al. 2014 | 48 | 0 | 0 |
| Barsukov et al. 2013 | 64 | 12 | 0 |
| Maluta et al. 2010 | 76 | 0 | 0 |

**Table S4.** Acute toxicity reported in seven studies

|  | **Selection** | | | | **Comparability** | **Outcome** | | |  |
| --- | --- | --- | --- | --- | --- | --- | --- | --- | --- |
| **Study** | **Representativeness of exposure** | **Selection of the non-exposed** | **Ascertainment of exposure** | **Outcome not present at start** |  | **Assessment** | **Follow-up length** | **Adequacy of follow-up** | **Overall score** |
| Wang et al. 2022 | ★ | - | ★ | ★ | - | ★ | ★ | ★ | 6 |
| Schem et al. 2022 | ★ | - | ★ | ★ | - | ★ | ★ | ★ | 6 |
| Lee et al. 2022 | ★ | - | ★ | ★ | ★★ | ★ | ★ | ★ | 8 |
| Ott et al. 2021 | ★ | - | ★ | ★ | - | ★ | ★ | ★ | 6 |
| Gani et al. 2021 | ★ | - | ★ | ★ | - | ★ | ★ | ★ | 6 |
| Shoji et al. 2017 | ★ | - | ★ | ★ | - | ★ | ★ | ★ | 6 |
| Gani et al. 2016 | ★ | ★ | ★ | ★ | ★ | ★ | ★ | ★ | 8 |
| Kato et al. 2014 | ★ | - | ★ | ★ | - | ★ | ★ | ★ | 6 |
| Barsukov et al. 2013 | ★ | - | ★ | ★ | - | ★ | ★ | ★ | 6 |
| Kang et al. 2011 | ★ | ★ | ★ | ★ | ★ | ★ | ★ | ★ | 8 |
| Maluta et al. 2010 | ★ | - | ★ | ★ | - | ★ | ★ | ★ | 6 |
| Rau et al. 1998 | ★ | - | ★ | ★ | - | ★ | ★ | ★ | 6 |

**Newcastle-Ottawa Quality Assessment Form for Cohort Studies**

Note: A study can be given a maximum of one star for each numbered item within the Selection and Outcome categories. A maximum of two stars can be given for Comparability.

**Selection**

1) Representativeness of the exposed cohort

a) Truly representative ***(one star)***

b) Somewhat representative ***(one star)***

c) Selected group

d) No description of the derivation of the cohort

2) Selection of the non-exposed cohort

a) Drawn from the same community as the exposed cohort ***(one star)***

b) Drawn from a different source

c) No description of the derivation of the non-exposed cohort

3) Ascertainment of exposure

a) Secure record (e.g., surgical record) ***(one star)***

b) Structured interview ***(one star)***

c) Written self-report

d) No description

e) Other

4) Demonstration that outcome of interest was not present at start of study

a) Yes ***(one star)***

b) No

**Comparability**

1) Comparability of cohorts on the basis of the design or analysis controlled for confounders

a) The study controls for age, sex and marital status ***(one star)***

b) Study controls for other factors (list) _________________________________ ***(one star)***

c) Cohorts are not comparable on the basis of the design or analysis controlled for confounders

**Outcome**

1) Assessment of outcome

a) Independent blind assessment ***(one star)***

b) Record linkage ***(one star)***

c) Self report

d) No description

e) Other

2) Was follow-up long enough for outcomes to occur

a) Yes ***(one star)***

b) No

Indicate the median duration of follow-up and a brief rationale for the assessment above:____________________

3) Adequacy of follow-up of cohorts

a) Complete follow up- all subject accounted for ***(one star)***

b) Subjects lost to follow up unlikely to introduce bias- number lost less than or equal to 20% or description of those lost suggested no different from those followed. ***(one star)***

c) Follow up rate less than 80% and no description of those lost

d) No statement

**Table S5.** Quality Assessment Studies according to Newcastle-Ottawa Quality Assessment


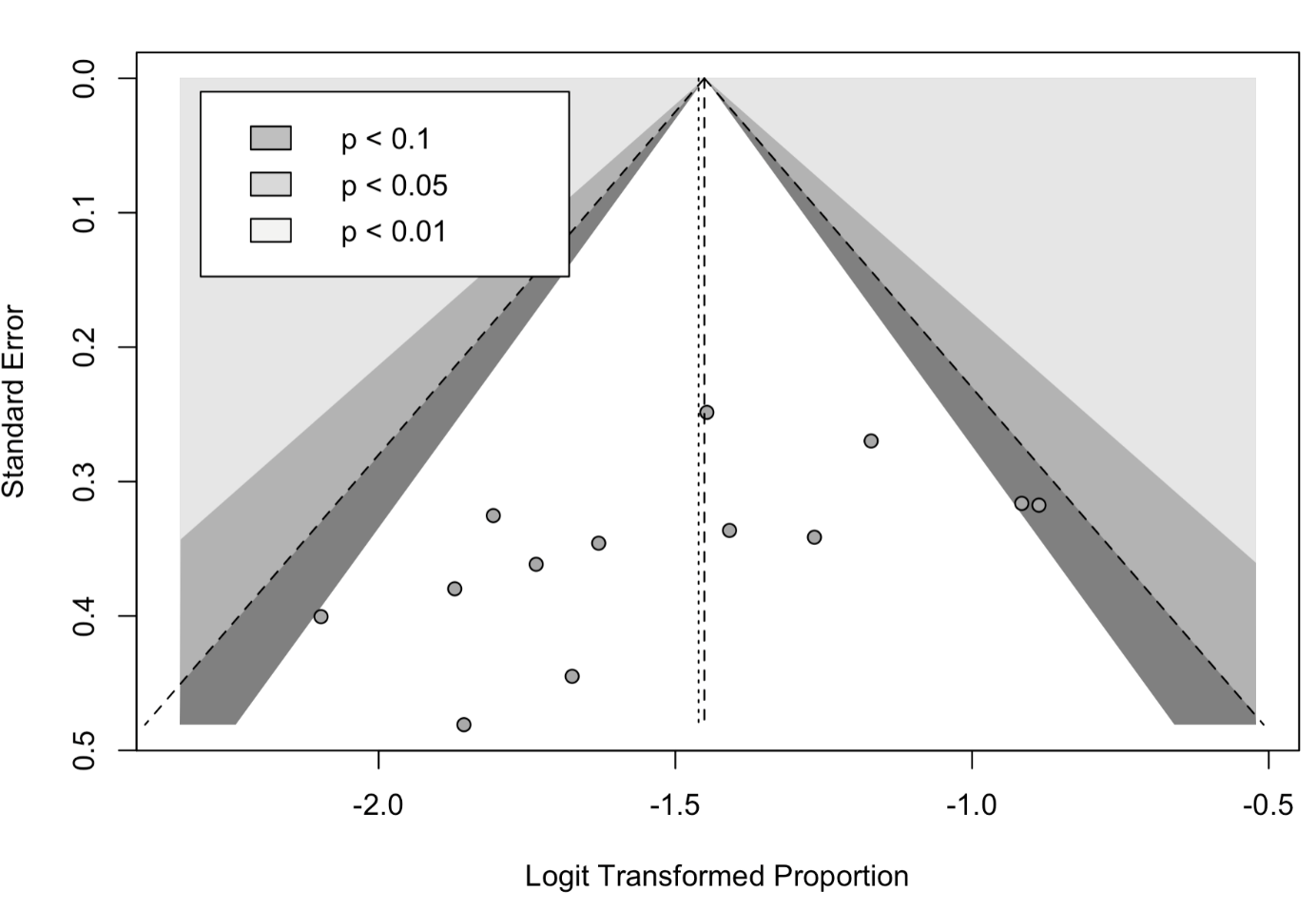


**Figure S8.** Funnel plot of studies reporting on pCR rate.

| **Number of studies** | **Certainty assessment** | | | | | | |  | **Effect** | | | **Certainty** | **Importance** |
| --- | --- | --- | --- | --- | --- | --- | --- | --- | --- | --- | --- | --- | --- |
|  | **Study design** | **Risk of bias** | **Inconsistency** | **Indirectness** | **Imprecision** | **Publication bias** | **Other considerations** |  | **N^o^ of events** | **N^o^ of individuals** | **Rate (95% CI)** |  |  |
| 12 | Observational studies | Serious^a^ | Not serious | Not serious | Low | Undetected | None |  | 144 | 778 | 0.19 (0.16-0.22) | ⊕⊕⊕◯  MODERATE | CRITICAL |
| ^a^ There was a lack of a control (non-exposed) group in all studies and a low level of comparability in the majority of the included studies | | | | | | | | | | | | | |

**Table S6.** GRADE assessment of results of meta-analysis for use of pCR in rectal cancer patients.
